# Rational dispensing of oral dosage forms of medicines to children at a teaching hospital in Sri Lanka

**DOI:** 10.1186/s12913-020-05246-x

**Published:** 2020-05-06

**Authors:** Abarna Nadeshkumar, Gitanjali Sathiadas, Shalini Sri Ranganathan

**Affiliations:** 1 Department of Pharmacy and Pharmaceutical Sciences, Faculty of Allied Health Sciences, University of Sri Jayewardenepura, Nugegoda, Sri Lanka; 2 Department of Paediatrics, Faculty of Medicine, University of Jaffna, Jaffna, Sri Lanka; 3 Department of Pharmacology, Faculty of Medicine, University of Colombo, Colombo, Sri Lanka

**Keywords:** Rational use, Oral dosage form, Dispensing, Children, Indicators

## Abstract

**Background:**

Good dispensing practice is vital for rational use of medicines. There are many paediatric specific challenges when maintaining good dispensing practices to children. Lack of age appropriate dosage forms, lack of medicines in strengths suitable for children, lack of palatable medicines, lack of expertise in paediatric pharmacy are few challenges faced when maintaining good dispensing practices to children. These challenges contribute to poor dispensing practices. Hence there is an urgent need to investigate whether oral dosage forms of medicines are dispensed rationally to children. The objective of this study was to describe the rational dispensing practice of oral dosage forms of medicines to children in a Teaching Hospital in Sri Lanka.

**Methods:**

A descriptive cross sectional study was conducted to assess the dispensing practice of 1800 oral dosage forms of medicines dispensed to children under the age of 12 years in two outdoor pharmacies over a period of 1 year using validated indicators. Required data were extracted from the prescriptions and by observation using a structured pre-tested observation sheet. Descriptive statistics and wherever relevant, chi square test were used in analysing the data.

**Results:**

Information on 1800 oral dosage forms was obtained from 1889 medicines dispensed to 727 children. Liquids were 52% [95% CI: 50–55%] of these oral dosage forms. Of the solid dosage forms, about one quarter required manipulation prior to administration such as splitting and dissolving or crushing the adult dosage form. None of the medicine packs or bottles had the patient name on the label.

**Conclusion:**

Dispensing practice of oral dosage forms of medicines to children has room for improvement.

## Background

The World Health Organisation (WHO) defines rational use of medicine as “patients receiving medications appropriate to their needs, in doses that meet their own individual requirements, for an adequate period of time, and at the lowest cost to them and their community” [1]. Good dispensing practices, which is defined as delivering an effective form of the correct medicine to the right patient, in the correct dosage and quantity, with clear instructions and in a package that maintains the potency of the medicine [2] is a core component in ensuring rational use of medicine.

Pharmacists face many obstacles in ensuring good dispensing practice in resource limited settings especially when dispensing to children due to limited resources and lack of expertise in paediatric pharmacy. For example, medicines not available in suitable size and dosage forms are the two major challenges faced by pharmacists every day when dispensing medicines to children in resource limited settings. These challenges are significantly high when disepensing oral dosage forms (ODFs) to young children since they cannot swallow tablets and capsules meant for adults. Everyday, pharmacists are compelled to dispense crushed tablets or tablets which had to be split by parents due to non availability of many medicines in sizes and dosage forms suitable for young children. Bio-availability, efficacy, quality, toxicity, palatability and acceptability of these manipulated adult dosage forms are questionable and become major hindrance to good dispensing practice and rational use of medicine [3, 4].

The current study is aimed to describe the dispensing practice of ODFs of medicines for a cohort of children in a Teaching Hospital using the newly developed and validated indicators in the Phase 1 of this large study [5].

## Methods

This was part of a large study, which investigated rational use of ODFs of medicines in children. This paper reports the dispensing practice of ODFs of medicines in children.

### Study design

Cross sectional descriptive study design as the study was aimed to describe the current practices [6]. As per the WHO recommendation encounters can be sampled prospectively or retrospectively [6]. However, in the absence of two essential elements namely (1) a method of selecting a random sample of patient encounters that took place within a defined period of time; and (2) the specific names and routes of administration of all drugs dispensed, the WHO methodology recommends prospective data collection. In our feasibility study we noticed that both elements were missing in the study setting.

### Study setting

The study was conducted from January 2017 for a year. The research was done in a Provincial General Hospital. This hospital is also the teaching hospital for the Faculty of Medicine in that province. Within the hospital, required data were collected from two study units: (1) Outpatient department (OPD) pharmacy where children who are not very sick with short term illness receive medicines, and (2) Paediatric clinic pharmacy where children with intermediate or long term illness who are on regular treatment receive medicines. Approximately 50–70 children attend both these places daily.

### Study sample

The study sample was the oral dosage forms of medicines dispensed to children in the OPD and clinic. The children were under the age of 12 years. Prescriptions dispensed in the above two study units to children under the age of 12 years, which had at least one ODF of medicine were used to obtain the required number of study sample.

### Sampling technique

Systematic sampling was used to select every other child but starting point (1^st^/2^nd^ child) was selected at random. Starting from the first child as per the clinic or OPD number order, prescription from every other child was screened to see whether it had at least one ODF of medicine. If not the prescriptions from the consequent children were screened until a prescription containing at least one ODF is identified. The process was repeated until 45 ODFs of medicines were surveyed. Each data collection time point was equalized to one health facility in that category. This method has been adopted by other researchers as well [7, 8]. Total sample size was 1800.

### Sample size

The WHO methodology on investigating drug use in health facilities recommends [6] that there should be at least 600 encounters ideally collected from 20 health facilities of same category (at least 30 encounters/ facility) for these types of studies. Since we were studying ODFs of medicines given to children under the age of 12 years, we equalized one ODF of medicine as one encounter. In the teaching hospital we had two categories of health facilities, OPD pharmacy and paediatric clinic pharmacy. Both OPD pharmacy and clinic pharmacy are stand alone health facilities for the entire province with no possibility of finding 20 facilities in the same category. World Health Organisation recommends that there should be at least 600 encounters included in a cross-sectional survey, with a greater number if possible. If 20 health facilities are included, as recommended, this means about 30 encounters per facility [6]. We decided to collect 45 encounters in 20 different time points over a year from each category (45 X 20 = 900 encounters). Each data collection time point was equalized to one health facility.

### Indicators

Indicators used to describe the practice of dispensing ODFs of medicines were developed and validated in the first phase of this large scale study [5] .The indicators are,

1. Percentage of instances where alternative oral dosage forms were dispensed
2. Percentage of oral dosage forms adequately labelled
3. Percentage of solid oral dosage forms irrationally manipulated by the pharmacist before dispensing
4. Percentage of solid oral dosage forms that needs manipulation before administering a single unit
5. Percentage of instances where oral dosage forms were dispensed with correct advice on storage

These indicators were converted to a data collection tool enabling the researchers to obtain the data from the prescriptions. This data collection tool was also pre-tested and inte-rater reliability was determined. Values for numerators and denominators to calculate the indicators were also pre-tested during this process [5].

### Data collection

Principal investigator personally collected the data prospectively from both OPD and clinic pharmacies in 20 time points over a period of 1 year. Required data to calculate the indicators were extracted from the prescription (OPD note or clinic book) while parents or guardians were waiting in the queue to collect medicines from the pharmacy and by observation of dispensing process. Informed written consent was obtained from parents/guardian as well as from the dispensing pharmacists.

### Data analysis

Data were analysed using Statistical Package for Social Sciences (SPSS23.0 IBM Corporation, NY). Descriptive statistics were used to calculate the indicators. Children to whom these ODFs were dispensed were categorized based on age as term new born (0–27 days), infants and toddlers (1 month to 23 months) pre-school children (2–5 years) and school children (6–11 years) [9]. Chi-square was used to determine significant difference in all indicators (indicators 1–5) between the OPD pharmacy and clinic pharmacy. Alpha level of .05 was used in statistical test and *P* < 0.05 was considered as significant.

## Results

Out of 924 medicines dispensed to 355 children at the clinic pharmacy 900 were ODFs [97.4%; 95% CI: 96–98%] with an average number of 2.54 ODFs [SD =1.43, range 1–12] per child and out of 965 medicines dispensed to 372 children at the OPD pharmacy 900 were ODFs [93.2%; 95% CI: 91–95%] with an average number of 2.42 ODFs [SD =0.77, range 1–5] per child (Table 1). Table 1 also presents the details of dosage forms dispensed at the two settings: Of the 900 ODFs, solid ODFs were dispensed to 79% of children in the clinic compared to 10% in OPD. This higher proportion in the clinic pharmacy was observed in all 3 age groups.

**Table 1:** Number of oral dosage forms dispensed to children and their basic characteristics

Setting	Number of ODFs (%)			Number of children (%)		
	Solid	Liquid	Total	Solid	Liquid	Total
**Clinic**						
**Age category**						
Infants and toddlers	196 (75)	67 (25)	263	63 (59)	43 (41)	106 (29.9)
Preschool children	267 (81)	62 (19)	329	99 (76)	31 (24)	130 (36.6)
School age children	299 (97)	09 (3)	308	116 (98)	3 (2)	119 (33.5)
**Total**	**762**	**138**	**900**	**278**	**77**	**355 (100)**
**OPD**						
**Age category**						
Term new-born	00 (0)	03 (100)	03	00 (0)	02 (100)	2 (0.5)
Infants and toddlers	03 (1)	336 (99)	339	01 (1)	140 (99)	141 (38)
Preschool children	16 (4)	391 (96)	407	7 (4)	155 (96)	162 (43.5)
School age children	74 (49)	77 (51)	151	29 (43)	38 (57)	67 (18)
**Total**	**93**	**807**	**900**	**37**	**335**	**372 (100)**
**Clinic**			**OPD**			
**Gender**						
Male	191 (53.8)				Male	185 (49.7)
Female	164 (46.2)				Female	187 (50.3)
**Total**	**355 (100)**				Total	**372 (100)**

### Indicator 1: percentage of instances where alternative ODFs were dispensed

Of the ODFs details received by the clinic pharmacist the dosage form details in the prescription was available only for 48.8% [95% CI: 45 -52%] ODFs. Alternative ODFs were dispensed only on 0.9%% [95% CI: 0.25–0.23%] of the instances.

Whereas 85% [95% CI: 82–87%] of the ODFs had the dosage forms in the prescriptions written by the OPD prescribers and only 0.7% [95% CI: 0.2–1.5%] of the instances alternative ODFS were dispensed. The difference noted between the two study units was not significant (*p* = 0.619).

### Indicator 2: percentage of oral dosage forms adequately labelled

Only 0.3% [95% CI: 0.07 -0.97%] were adequately labelled by the OPD pharmacists and none by the clinic pharmacists.

All the ODFs dispensed by both pharmacies had the frequency and duration on the label. Only 15% of the ODFs dispensed by the OPD pharmacy and 43% of the clinic pharmacy had the drug name. Only 0.3% of the dispensed by the OPD had the drug name and none of the ODFs dispensed by the clinic pharmacy had the drug name on the label. The difference noted between the two study units was not significant (*p* = 0.083).

### Indicator 3: percentage of solid oral dosage forms irrationally manipulated by the pharmacist before dispensing

None of ODFS were irrationally manipulated by the OPD pharmacist before dispensing, 2.8% [95%CI: 1.8–4.1%] were irrationally manipulated by the clinic pharmacist and dispensed. They were crushed and made into powder and packed as sachet packets. They were dispensed more to children under the age of 2 years. The difference noted between the two study units was not significant (*p* = 0.103).

### Indicator 4: percentage of solid oral dosage forms that needs manipulation before administering a single unit

Of 762 solid ODFs dispensed by clinic pharmacy 22% [95% CI: 19 - 25%] needed manipulation before administering single unit. Out of 93 solid ODFs dispensed by the OPD pharmacy 25% [95% CI: 16–35%] needed manipulation before administration. The difference noted between the two study units was not significant (*p* = 0.578).

### Indicator 5: percentage of instances where oral dosage forms were dispensed with correct advice on storage

Though storage conditions are important for all the dosage forms we concentrated on reconstituted antibiotic suspensions. Of the antibiotic suspension (*n* = 166) dispensed in both pharmacies storage advice were given only to 10% [95% CI: 6–15%]. The difference noted between the two study units was not significant (*p* = 0.874).

Table 2 gives the measurement of indicators.

**Table 2:** Summary of indicator measurements

No	Indicator	Ideal (%)	Clinic (%)	OPD (%)	*p* value
01	Percentage of instances where alternative oral dosage forms were dispensed	0	0.9	0.7	0.619
02	Percentage of oral dosage forms adequately labelled	100	0	0.3	0.083
03	Percentage of solid oral dosage forms irrationally manipulated by the pharmacist before dispensing	0	2.8	0	0.103
04	Percentage of solid oral dosage forms which needs manipulation before administering a single unit	0	22	25	0.578
05	Percentage of instances where oral dosage forms were dispensed with correct advice on storage	100	8.3	9.7	0.874

### Resources and infrastructure

No reference sources required for paediatric dispensing was available in the pharmacies. Calculators and refrigerators were available in both pharmacies. Space was available for pharmacists to do some form of extemporaneous preparations or manipulation of adult dosage forms before dispensing in both pharmacies. However, there was no written standard operating procedures for these processes in both these pharmacies.

## Discussion

Several studies have documented irrational use of medicines in children in resource limited settings [10–12]. Though ODFs are the most commonly used dosage forms for children and good dispensing practice is an essential prerequisite for rational use of medicine in children, to our best of knowledge, there had been no published studies in the literature reporting dispensing practice of ODFs in children in resource limited settings.

Though our inclusion criterion was ODFs dispensed to children under the age of 12 years, majority of study sample happened to be children under the age of 6 years. This is a natural trend as infectious diseases are more in this age group and parents are more anxious about this age group than the older children. This in a way gave us rich data as majority of children in this age group will not be able to use intact solid ODFs.

Though tablets and capsules are not considered suitable for children under the age of 2 years [9], we reported that 26% of children in this age group received tablets or capsules. Tablets and capsules have been given to children under the age of 2 years which is not recommended. Tablets and capsules would have been tested on children under the age of 2 years. Parents would have definitely manipulated (split/ crushed and mixed with food or other liquids/opened the capsules) tablets and capsules before administering. The efficacy, bio availability and safety of tablets and capsules after manipulation is questionable. This leads to irrational use of medicine in children.

Crushing tablets increases medicine potency and manipulating controlled release preparations and enteric coated tablets prior to administration can significantly change the medicine bioavailability and affect clinical response [13].

A study has shown that only 3 out of the 11 segments obtained from splitting the tablets by razor blade passed an adapted USP uniformity test [14]. McDevitt and colleagues examined the accuracy of tablet splitting by volunteers. Of the split tablet portions, 41.3% deviated by more than 10% from ideal weight, and 12.4% of the portions deviated by more than 20%. After this experience on splitting, 77.2% of the volunteers have confirmed their willingness to pay more for a standard tablet of the lower strength [15]. Our study found that almost one quarter of the dispensed dosage forms needed manipulation before administration.

Adequately labelled has been defined by the WHO as ‘drug packages containing at least patient name, drug name and when the drug should be taken’ [6]. The study highlights that the percentage of ODFs adequately labelled was very minimal which appears to be much lower than the other developing countries [7, 16]. If the standard of adequately labelling practice does not improve it always have the potential to cause medication errors. In case of anti-bacterial agents, practices like improper reconstitution technique, measuring without shaking the suspension and not storing according to the instructions given by the manufacturer would cause under or over dosing which in turn will cause anti-bacterial resistance or potential health risk to the child. Even in a busy pharmacy, pharmacists should not neglect advising parent /guardian on administration and storing of anti-bacterial suspensions.

Reasons for this substandard dispensing practice could be related multifold: unavailability of the suitable doses for the medicines may be the reason for this manipulation. High number of patients and lack of staff may be the reason for not advising the patients on storage.

None of the indicators were significantly different between the two study units. This could be explained by the fact that the challenges such as non-availability of child friendly formulations, high pharmacist: patient ratio and lack of paediatric pharmacists are common to both study units. In addition, pharmacists are not permanently assigned to either OPD or clinic pharmacy, but rotate from one setting to the other based on administrative decisions. Thus, opportunities for pharmacists to professionally develop setting-specific dispensing practice are limited.

There are some limitations in our study: for example, the study was restricted to two categories of health facilities in a single large hospital. This may not be a representative sample of similar health facilities in the country. However we feel the issues observes in the hospital which is a large teaching hospital should be considered as important issues. Hospitals in the similar level or lower level will have same or higher degree issues like the issues we observed in study hospital. Since we collected at 20 time points in one study unit, it can be argued that the same problems will be recurrently seen. However, our objective was to measure the extent of irrational dispensing practice of ODFs of medicines in children. Though the problems are similar we were able to give a numerical value for the problem. Storage plays an important role especially in reconstituted antibacterial suspension. Since advice on storage is more important to reconstituted antibacterial suspension, to calculate indicator 5 (Percentage of instances where oral dosage forms were dispensed with correct advice on storage) we considered only the reconstituted antibacterial suspension dispensed.

## Conclusion

Dispensing practices of ODFs of medicines to children has room for improvement in the study Teaching Hospital. We assume that there are several reasons for irrational use of oral dosage forms in children. We recommend that at least for important group of medicines child should be prescribed with child friendly ODFs. Continuous availability of child friendly ODFs will reduce the extent of this problem. In addition, pharmacists should be provided continuous professional education on paediatric pharmacy skills such as forecasting requirements, labelling, storage, communicating with parents and providing information.

## Data Availability

This is part of a large study hence the dataset generated and/or analysed during the current study are not publicly available but are available from the corresponding author on reasonable request.

## Abbreviations

ODF: Oral dosage form
WHO: World Health Organisation
OPD: outpatient department
